# Supplementary material for: A Derivative of Butyric Acid, the Fermentation Metabolite of Staphylococcus epidermidis, Inhibits the Growth of a Staphylococcus aureus Strain Isolated from Atopic Dermatitis Patients
Source: Toxins (Basel). 2019 May 31;11(6):311. doi: 10.3390/toxins11060311 (PMC6628397; doi:10.3390/toxins11060311)
Supplement: Supplementary file 1 [file toxins-11-00311-s001.pdf]

Supplementary Materials: A Derivative of Butyric Acid, the Fermentation Metabolite of *Staphylococcus epidermidis*, Inhibits the Growth of a *Staphylococcus aureus* Strain Isolated From Atopic Dermatitis Patients

Supitchaya Traisaeng, Deron Raymond Herr, Hsin-Jou Kao, Tsung-Hsien Chuang and Chun-Ming Huang *

1. Reduction of IL-6 Production by BA-NH-NH-BA

In Figure 3c, we showed that AD *S. aureus* induced IL-6 production in mouse skin. Application of BA-NH-NH-BA onto AD *S. aureus*-colonized skin reduced IL-6 production (Figure 6d). Since BA-NH-NH-BA exhibited anti-*S. aureus* activity (Figure 5b,c), we next examined whether the anti-inflammatory activity of BA-NH-NH-BA is mediated by the growth suppression of AD *S. aureus*. Treatment of human keratinocyte (KERTr) cells with heat-killed AD *S. aureus* lysate for 24 h resulted in a marked increase in IL-6 production. This increase was not observed with PBS or BA-NH-NH-BA treatment alone (Figure S4). By contrast, the production of IL-6 induced by AD *S. aureus* lysate was considerably reduced when skin was applied with BA-NH-NH-BA (Figure S4). This result suggests that HDAC inhibition (Figure 6a) by BA-NH-NH-BA directly regulates cytokine production.

2. The Level of IL-6 and Number of AD *S. aureus* in the HDAC-Depleted Mice

We next investigated if *in vivo* inhibition of HDAC influenced the production of IL-6 in skin and the skin colonization of AD *S. aureus*. HDACs in mice were depleted by administration of 3,3'-diindolylmethane (DIM) according to a published protocol [1]. Skin wounds in mice treated with/without DIM were topically applied with AD *S. aureus* (10^8 CFU/10 µl) bacteria for 24 h. As shown in Figure S5a, the level of IL-6 in wounded skin of mice treated with DIM (840 ± 100 pg/mL) was lower than that of wounded skin from mice without DIM treatment (2458 ± 86 pg/mL). However, there was no difference in the number of AD *S. aureus* colonized in skin wounds between two groups of mice treated with/without DIM (Figure S5b,c). These results indicate that depletion of HDACs by DIM caused a decline in IL-6 production without changing the skin colonization of AD *S. aureus*.

3. Materials and Methods

3.1. Keratinocytes Treated with Bacterial Lysates in the Presence or Absence of BA-BNH-NH-BA

KERTr cells (CCD 1106 KERTr (ATCC® CRL-2309™) were grown in keratinocyte-serum-free medium (SFM) (Gibco-BRL) supplemented with bovine pituitary extract, recombinant epidermal growth factor and 1% of penicillin. Cells (5×10^4 cells/mL) were seeded in a culture dish and cultured for 3 d before treatments of PBS, AD S. aureus lysate (100 µg), BA-NH-NH-BA (100 µM), or AD S. aureus lysate with BA-NH-NH-BA) for 24 h. AD S. aureus was killed at 80 °C for 15 min. The lysate was obtained by resuspending bacterial pellets with PBS. The level of IL-6 in culture media was measured by ELISA using a human IL-6 ELISA kit (R&D systems, Minneapolis, MN, USA).

3.2. Administration of DIM into Mice

Two doses of DIM (40 mg/kg body weight, Sigma Chem-Impex, Wood Dale, IL, USA), which was dissolved by 2% dimethyl sulfoxide (DMSO) in corn oil in a total volume of 100 µL, were intraperitoneally administered into ICR mice [1]. The second dose of DIM was given 24 h after first administration. Mice without administration of DIM serve as a control group. A 1 cm wound was made on the dorsal skin of mice and AD *S. aureus* (10^8 CFU/10 µL) bacteria were topically applied onto the wound 30 min after

administration of the second dose of DIM. 24 h after application of AD *S. aureus*, the number of bacteria and the level of IL-6 in skin wound were measured as described in Materials and Methods in the main text of manuscript.

3.3. GC Analysis

BA-NH-NH-BA (4 mM) was dissolved in PBS and stored at 4 °C for six months and detected by ethyl acetate liquid-liquid extraction and saturation with sodium chloride followed by GC analysis using an Agilent 5890 Series II GC [2].

3.4. Terminal Deoxynucleotidyl Transferase dUTP Nick End Labeling (TUNEL) Assay

To examine the cytotoxicity of BA-NH-NH-BA, dorsal skin of ICR mice were topically applied with BA-NH-NH-BA (4 mM) or PBS for 24 h. The skin was excised, immersed and fixed in 10% formalin. The tissue sections of skin were cut with a thickness of 3 μm for TUNEL staining (R&D systems, Minneapolis, MN, USA). To quantify the TUNEL-negative (nuclear, blue staining) and -positive (nuclear, brown staining) cells, a total of at least 3 randomly selected stained images with more than 50 cells were counted.

CNTGCCAGTCGAGCGAACGGACGAGAAGCTTGCTTCTCTGATGTTAGCGGCGGAC
GGGTGAGTAACACGTGGATAACCTACCTATAAGACTGGGATAACTTCGGGAAACCG
GAGCTAATACCGGATAATATTTTGAACCGCATGGTTCAAAAGTGAAAGACGGTCTT
GCTGTCACTTATAGATGGATCCGCGCTGCATTAGCTAGTTGGTAAGGTAACGGCTTA
CCAAGGCAACGATGCATAGCCGACCTGAGAGGGTGATCGGCCACACTGGAACTGA
GACACGGTCCAGACTCCTACGGGAGGCAGCAGTAGGGAATCTTCCGCAATGGGCG
AAAGCCTGACGGAGCAACGCCGCGTGAGTGATGAAGGTCTTCGGATCGTAAAACT
CTGTTATTAGGGAAGAACATATGTGTAAGTAACTGTGCACATCTTGACGGTACCTAA
TCAGAAAGCCACGGCTAACTACGTGAACCCCCAAAAAAAAAATTAGC

Figure S1. 16S ribosomal RNA (rRNA) sequence of AD *S. aureus* using the 16S rRNA 27F and 534R primers.

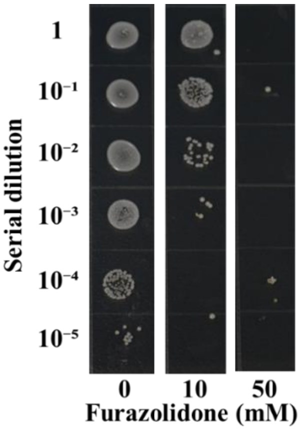

Figure S2. Inhibition of *S. epidermidis* growth by furazolidone. *S. epidermidis* (10^7 CFU) was cultured with 0, 10, and 50 mM of furazolidone for 24 h. The agar plate shows the serial dilution of *S. epidermidis* for each concentration. 50 mM furazolidone resulted in complete lethality of *S. epidermidis*.

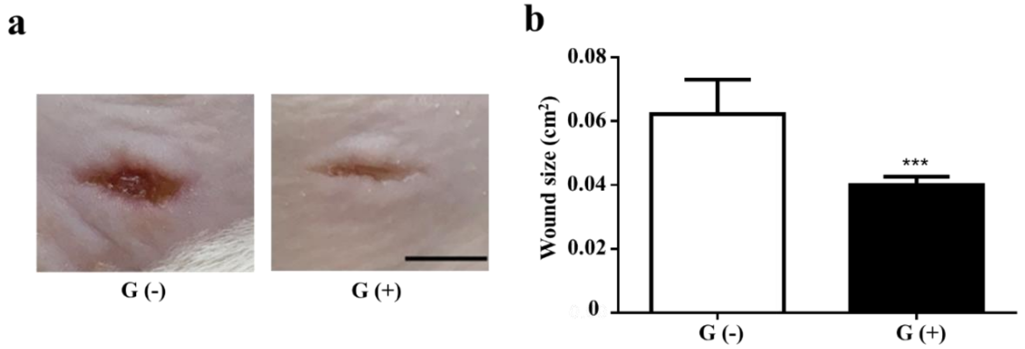

Figure S3. Effects of glycerol fermentation by *S. epidermidis* on wound healing in vivo. (**a**) Wound photographs of *S. epidermidis* (10^7 CFU) and AD *S. aureus* (10^7 CFU) with (G+)/without (G−) 2% glycerol treatments for 3 d (scale bar, 5 mm). (**b**) Wound size (cm^2) was quantified from the photographs. Data shown are mean ± SE. *** $p < 0.001$ (two-tailed *t*-test).

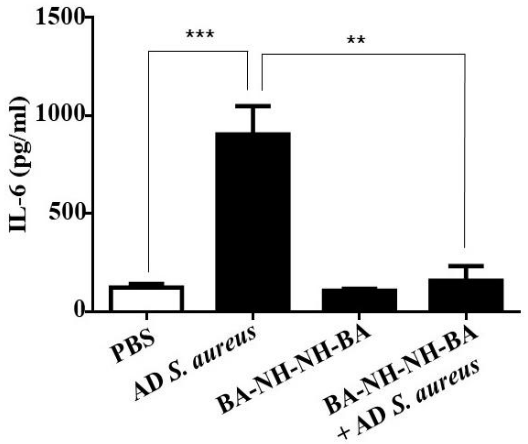

Figure S4. Levels of the pro-inflammatory IL-6 cytokine in KERTr cells treated with PBS, lysate of AD *S. aureus* (100 μg), BA–NH–NH–BA (0.1 mM), and lysate of AD *S. aureus* plus BA–NH–NH–BA for 24 h. The IL-6 was quantified by IL-6 ELISA kits. ** $p < 0.01$, *** $p < 0.001$ (two-tailed *t*-test).

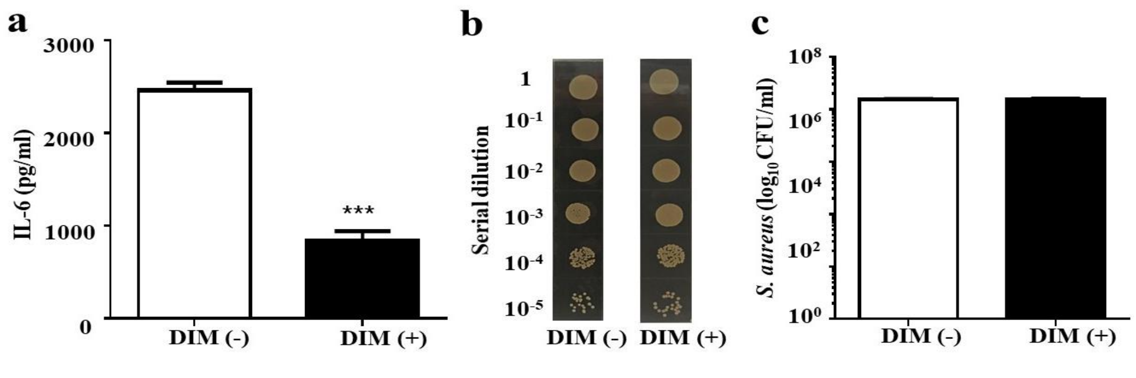

Figure S5. Level of IL-6 and bacterial growth in AD *S. aureus*-colonized skin wounds of mice pretreated with DIM. HDACs in ICR mice were depleted by DIM as described in Materials and Methods. AD *S. aureus* (10^8 CFU) bacteria were topically applied onto the wounds of mice-treated with (DIM+) or without (DIM-) DIM. Twenty-four h after bacterial application, the level of IL-6 was detected by ELISA (**a**). Bacterial CFUs in the skin wounds were enumerated by plating serial dilutions ($1:10^1$–$1:10^5$) of the skin homogenate on TSB plate**s** (**b**) and the number (log_{10} CFU/mL) of AD *S. aureus* (c) were measured. Data shown are mean ± SE. *** $p < 0.001$ (two-tailed *t*-test).

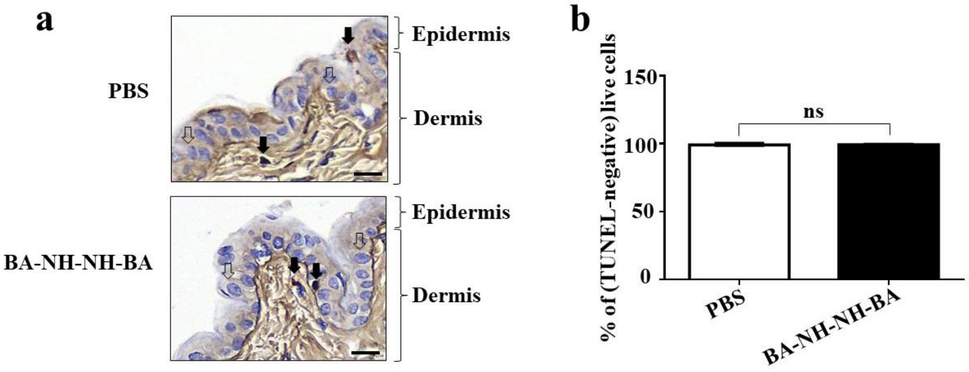

Figure S6. No significant cytotoxic effect of BA–NH–NH–BA. (**a**) Histology (TUNEL staining) of mouse skin (epidermis and dermis) 24 h after topical application of 4 mM BA–NH–NH–BA or PBS. Scale bars: 30 μm. (**b**) Percentage of (TUNEL-negative) live cells in skin applied with BA–NH–NH–BA or PBS was quantified. Data shown are mean ± SE. ns, not significant.

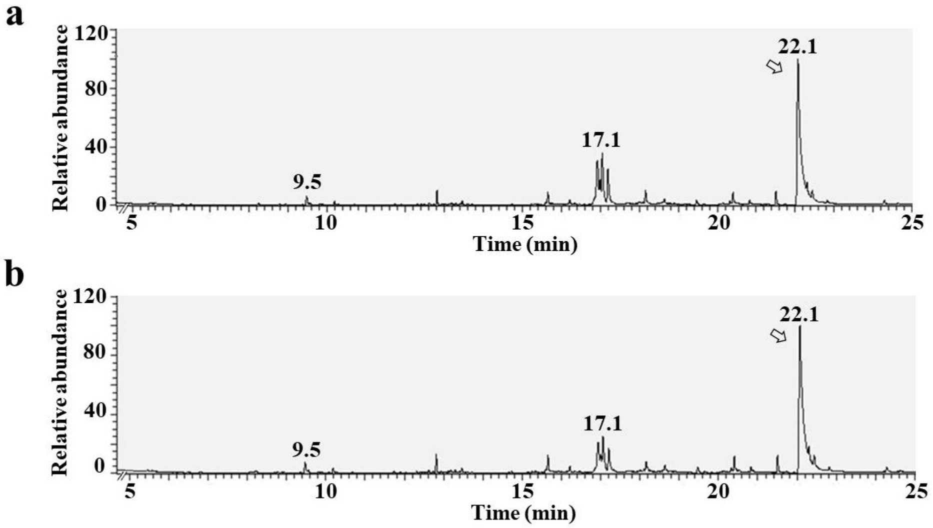

Figure S7. Stability of BA–NH–NH–BA by GC analysis. BA–NH–NH–BA was detected by GC after fresh preparation (**a**) six months of storage (**b**) at 4 °C. BA–NH–NH–BA with a retention time of 22.1 min (arrows) was detected.

References

1. Busbee, P.B.; Nagarkatti, M.; Nagarkatti, P.S. Natural indoles, indole-3-carbinol (I3C) and 3, 3′-diindolylmethane (DIM), attenuate staphylococcal enterotoxin B-mediated liver injury by downregulating miR-31 expression and promoting caspase-2-mediated apoptosis. *PloS ONE* **2015**, *10*, e0118506.
2. Kao, M.S.; Huang, S.; Chang, W.L.; Hsieh, M.F.; Huang, C.J.; Gallo, R.L.; Huang, C.M. Microbiome precision editing: Using PEG as a selective fermentation initiator against methicillin-resistant Staphylococcus aureus. *Biotech. J.* **2017**, *12*, doi:10.1002/biot.201600399.
